# An Interesting Case of Euthyroid Graves’ Ophthalmopathy, With Negative Thyroid-Stimulating Hormone Receptor Antibodies

**DOI:** 10.7759/cureus.19015

**Published:** 2021-10-24

**Authors:** Zunera Moeen, Ammar M Aliuddin, Taylor Gray Wlazlo, Hira Majid, Swapna Kolli

**Affiliations:** 1 Internal Medicine, Texas Tech University Health Sciences Center, Odessa, USA; 2 Internal Medicine, University of Cincinnati College of Medicine, Cincinnati, USA; 3 Obstetrics and Gynaecology, Texas Tech University Health Sciences Center, Lubbock, USA; 4 Internal Medicine, Dow University of Health Sciences, Karachi, PAK

**Keywords:** thyroid ophthalmopathy, endocrinology and diabetes, thyroid eye disease, stellwag sign, teprotumumab, thyroxine (t4), thyroid pathology, graves' orbitopathy, euthyroid

## Abstract

Thyroid eye disease (TED), also known as Graves' orbitopathy or ophthalmopathy (GO) or Graves' eye disease, is an autoimmune condition of the retroocular tissues associated with Graves’ disease. In isolated GO, the patient can present without thyroid hormone dysfunction or systemic symptoms of Graves’ disease, in which case it is called euthyroid Graves’ ophthalmopathy (EGO). It is very rare for this condition to have negative thyroid-stimulating hormone receptor (TSHR) autoantibodies, and we present such a rare case of a young female, who had progressive bilateral vision loss, intermittent left-sided retroocular headache, and severe bilateral proptosis. The patient was diagnosed with EGO based on multidisciplinary consults, diagnostic orbital magnetic resonance imaging (MRI) results, and a good response to treatment with intravenous steroids. Later, the patient was followed as an outpatient and treated with thyroid orbitopathy-specific immunotherapy with teprotumumab. The patient’s response to teprotumumab was excellent and caused significant improvement in visual acuity, proptosis, and chemosis. This adds valuable literature to the medical field and gives insight to clinicians to consider the diagnosis of GO even with seronegative TSHR autoantibodies and euthyroid hormone status. It also adds to the understanding of the complex pathophysiology of this rare condition.

## Introduction

Ophthalmopathy is one of the most distinguishable features of Graves’ disease. It is depicted by eyelid retraction, diplopia, exophthalmos, lid lag, restrictive extraocular myopathy, and optic neuropathy [1]. Only 10% of Graves' orbitopathy (GO) patients present without typical symptoms of thyroid hormone abnormality, and such isolated presentation of ophthalmopathy is known as euthyroid Graves’ ophthalmopathy (EGO). Even though thyroid-stimulating hormone receptor (TSHR) autoantibodies are thought to be essential in the pathophysiology of GO, in rare cases, they are not detected in the serum, which highlights the paradox in the disease pathophysiology versus its diagnosis. We present such a unique case of EGO with seronegative TSHR autoantibodies. In approximately 20% of cases, orbitopathy precedes the onset of hyperthyroidism. The duration between orbitopathy and onset of thyroid symptoms may be a few weeks up to a few years, requiring close monitoring of these patients.

## Case presentation

A 25-year-old female with uncontrolled diabetes presented to our inpatient service with a eight-month history of gradual bilateral vision loss and intermittent left-sided retroocular headache. Ophthalmic examination showed bilateral proptosis, retrobulbar pain, conjunctival redness, lacrimation, and decreased visual acuity (VA). Due to classic ocular features of Graves’ disease, thyroid function tests T3, T4, and thyroid-stimulating hormone (TSH) including serological tests like anti-TSHR, thyroid peroxidase antibody, thyroid-stimulating antibody, and thyroglobulin antibodies were also performed. Results of all the tests came back normal. Values are referenced in Table 1.

**Table 1:** Tabulated presentation of serum thyroid hormone levels and serology levels in the presented patient. T4: L-thyroxine; TSH: thyroid-stimulating hormone; T3: 3, 3′, 5-L-triiodothyronine; anti-Tg Ab: anti-thyroglobulin antibody; anti-TPO Ab: anti-thyroid peroxidase antibody; anti-TSI Ab: anti-thyroid-stimulating antibody; anti-TSHR Ab: anti-thyroid-stimulating hormone receptor/thyrotropin antibody; ng/dL: nanogram per deciliter; mlU/L: milli-international units per liter; IU/mL: international units per milliliter; IU/L: international units per liter *Not available

Test	Initial results	Follow-up results	Reference range	Units
Free T4	1.10	1.03	0.07-1.40	ng/dL
TSH	1.98	0.95	0.34-5.30	mlU/L
Total T3	NA*	138	80-200	ng/dL
Anti-Tg Ab	<0.9	NA*	0.0-4.0	IU/mL
Anti-TPO Ab	1.5	NA*	0.0-9.0	IU/mL
Anti-TSI Ab	89	NA*	$\leq$122	%
Anti-TSHR Ab	1.6	NA*	0.0-1.75	IU/L

**Figure 1:**
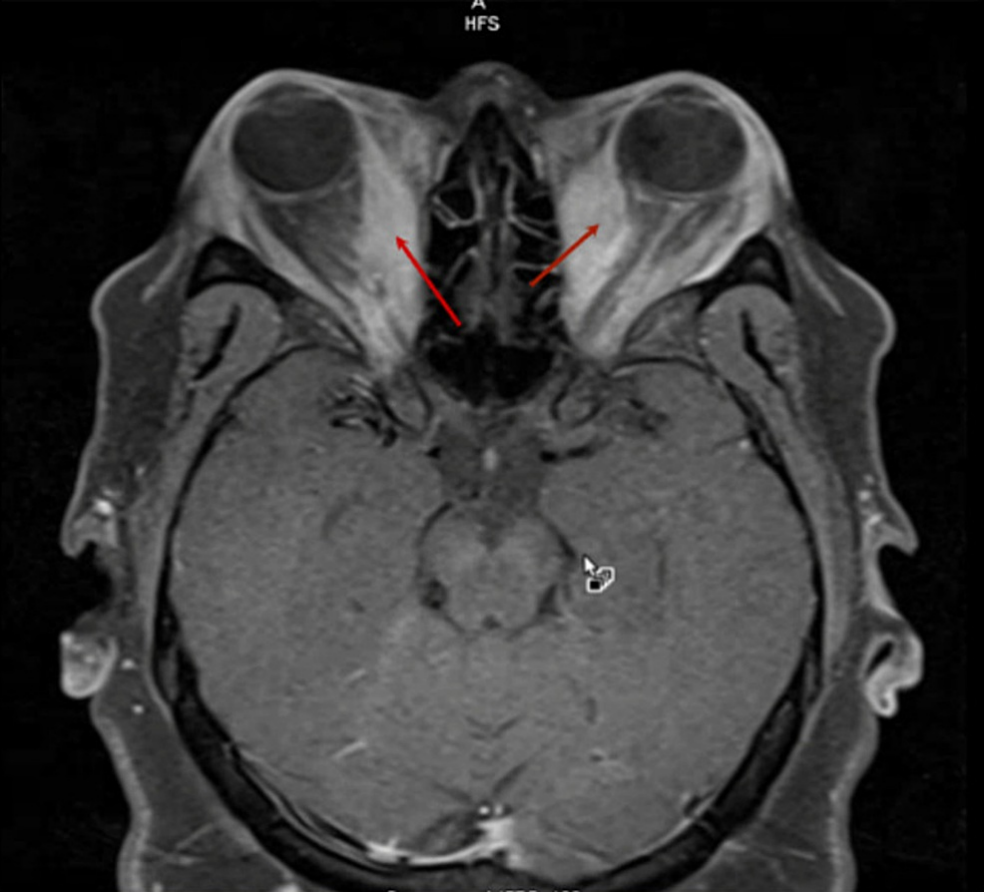
Orbital MRI (axial view) showing significant enlargement of extra-ocular muscles (bilateral medial recti indicated by red arrows), giving a sign known as "Coca-cola bottle" appearance. MRI, magnetic resonance imaging

Due to these concerning findings on the eye examination, an ophthalmology consult was requested. Eye examination showed the ophthalmometer-measured diameter of the eye to be 30 mm compared to normal of 14-18 mm. Fundoscopic examination revealed optic atrophy. Orbital and brain magnetic resonance imaging (MRI) was performed to rule out other causes of proptosis like a mass and to determine the extent of ocular disease. Results of orbital MRI showed bilateral enlargement of the medial recti muscles compressing both the optic nerves (Figure 1).

**Figure 2:**
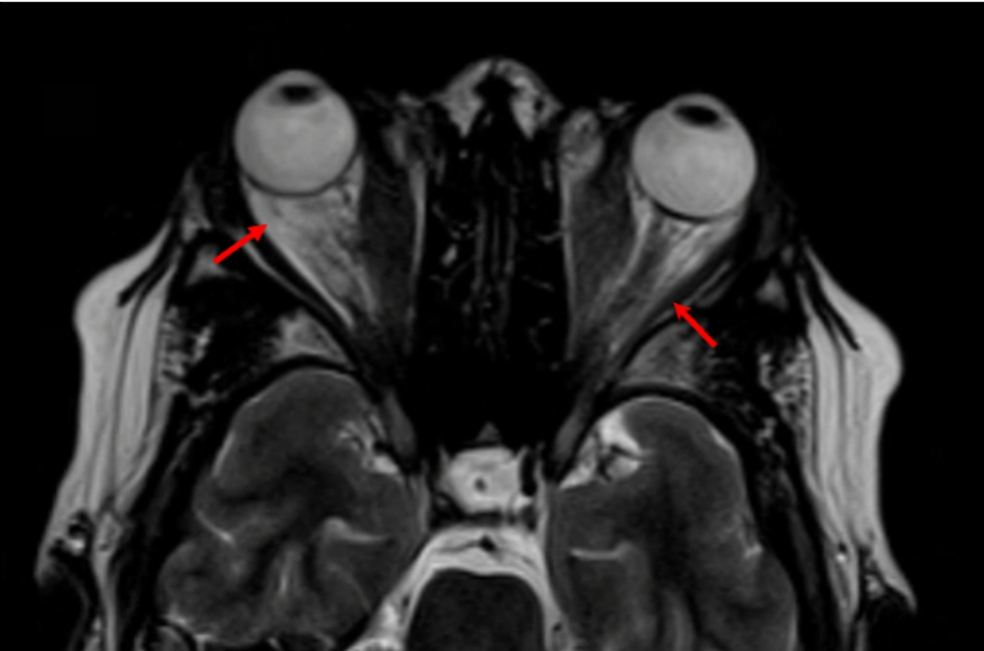
Orbital MRI (axial view) showing bilateral proptosis. There is a generalized increase in the volume of orbital fat (indicated by red arrows). MRI, magnetic resonance imaging

**Figure 3:**
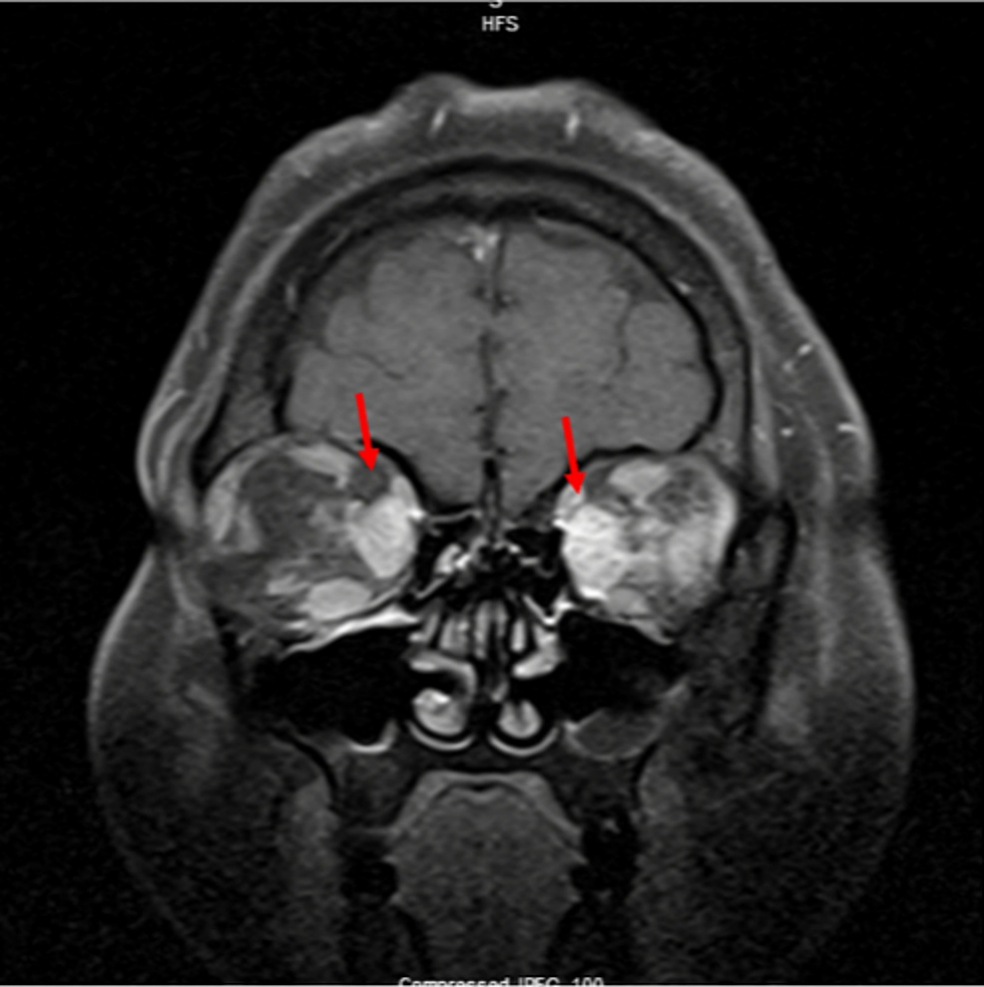
Orbital MRI (coronal view) shows another view of bilateral proptosis and ocular muscle hypertrophy indicated by red arrows. MRI, magnetic resonance imaging

The right inferior rectus muscle was enlarged with notable bilateral orbital fat stranding and extensive edema (Figures 2, 3).

Brain MRI did not show any mass, space-occupying lesions of the brain, hydrocephalus, or infarction. With these peculiar findings of the extraocular muscles and orbit, diagnosis of EGO was made with consensus by endocrinology and ophthalmology. The patient was started on pulse dose intravenous steroids for three days. Her proptosis, eye swelling, and VA improved by the second day of steroid administration. Upon discharge, the patient was transitioned to oral steroids. Since the patient had uncontrolled diabetes and with this new diagnosis of EGO, she was advised to closely follow with the institutional outpatient endocrinology clinic. Considering her uncontrolled diabetes status, she was transitioned from steroids and offered the treatment targeted for thyroid orbitopathy, i.e., immunotherapy with teprotumumab. The patient received seven infusions of teprotumumab and showed a promising response. Significant improvement in VA, proptosis, and chemosis was noticed upon follow-up and the overall clinical activity score was improved.

## Discussion

TED is a rare, debilitating autoimmune disease with an incidence of 2.9-16.0 cases per 10,000 population per year [1]. The pathogenesis of GO is thought to be due to TSHR autoantigen expression in adipocytes and fibroblasts along with T cell activation. This leads to an increased accumulation of hydrophilic glycosaminoglycans and a resulting increase in the volume of both retroocular connective tissue and extraocular muscles [2]. IGF-1 receptor (IGF-1R) autoantigen expression is another proposed mechanism for the stimulation of orbital fibroblasts. Activation of this receptor may act in parallel or conjunction with the TSHR activity in this disease via downstream enzyme activation or downstream post-receptor signaling [3].

Various classification systems are present to describe the clinical features of GO. In 1969, NO SPECS Classification was introduced. It is an acronym for No physical signs or symptoms, Only signs, Soft tissue involvement, Proptosis, Extraocular muscle signs, Corneal involvement, and Sight loss. In 1989, the Clinical Activity Score (CAS) was devised, which is a tool used not only to assess predictive certainty for the diagnosis but to track the treatment response of GO [4]. This score is based on classic signs of inflammation (pain, redness, swelling, and impaired function). If overall CAS is ≥4, then the positive predictive value is 80% and the negative predictive value is 64%. In our patient, CAS was 4. After the use of teprotumumab, her CAS improved to 0. Currently, VISA classification (Vision, Inflammation, Strabismus, and Appearance) and the European Group of Graves’ Orbitopathy (EUGOGO) Classification are also widely used. Both stem from NO SPECS and CAS classifications and include indicators for signs of activity and the degree of severity, and therefore guide clinicians on the treatment modality to be used [4-6]. Details of the classification systems extend beyond the scope of the current discussion. Other conditions that can mimic the eye findings in EGO include orbital tumors, histiocytosis, orbital myositis, Cushing's syndrome, myasthenia gravis, cavernous carotid fistula, and IgG4-related disease. All of these should be ruled out by thorough history, examination, imaging, and labs [7,8]. Fifty percent of EGO patients report dryness and grittiness in the eye, hypersensitivity to light, excess lacrimation, diplopia, and pressure behind the eyes. Common signs are edema, erythema of the periorbital tissues and conjunctivae, proptosis, Dalrymple sign (upper eyelid retraction), von Graefe sign (retarded descent of upper lid at downward gaze), and Stellwag sign (infrequent blinking). About 3-5% have severe disease with intense pain, inflammation, and sight-threatening corneal ulceration or compressive optic neuropathy. About 70% of adult patients with Graves’ hyperthyroidism have an extraocular-muscle enlargement on MRI or computerized tomography (CT) scans. The asymmetric bilateral eye disease is common and confirmed on orbital imaging, but clinically unilateral EGO occurs occasionally. Upon examination, Dalrymple, von Graefe, and Stellwag signs were all present in our patient [9].

Cessation of smoking is recommended in the primary, secondary, and tertiary prevention of EGO. In mild disease, local therapy with artificial tears, dark glasses, nocturnal taping of the eye, and prims can control symptoms. Many patients experience spontaneous improvement or resolution of symptoms with these measures. The best management of EGO is through the collaborative effort of endocrinology and ophthalmology to preserve patient’s vision and improve quality of life. Hormonal treatment should be appropriately given if thyroid hormone levels are deranged. The most common regimen is to give high-dose steroids in the first month with subsequent gradual tapering over the next two months. Immunomodulator therapy with agents like rituximab and cyclosporine is also studied for treatment, with moderate benefit [10].

A new immunotherapeutic agent, teprotumumab, is a human monoclonal antibody that targets IGR-1R and suppresses both the individual effects of IGF-1R and the complex’s effects of IGF-1R/TSHR. This leads to an overall decrease in the production of hyaluronic acid by orbital fibroblasts, therefore deterring the disease-causing immune response in active EGO. With the use of teprotumumab, this patient underwent significant improvement in disease signs and symptoms, with improvement in CAS [11]. The OPTIC trial, a multicenter study conducted over 24 weeks' period, showed that teprotumumab administered once every three weeks was significantly more effective than placebo to decrease extraocular muscle volume (improved proptosis and diplopia) and orbital fat (improved proptosis), in moderate-to-severe, active TED. If the disease is severe but active, treatment with glucocorticoids/immunotherapy or orbital decompression is performed. Once conservative treatment inactivates the disease, rehabilitative (extraocular muscle or eyelid) surgery or orbital decompression is often needed for residual eye changes, ideally after three or more months of observation [12].

## Conclusions

This case illustrates the unique situation in which GO can occur while being seronegative for thyroid autoantibodies and with euthyroid hormone status. Diagnosing GO is a complex process requiring meticulous review of signs, symptoms, and imaging results while excluding other causes of proptosis such as orbital mass/tumors. It emphasizes the fact that relying on thyroid hormone and autoantibody levels alone is not always adequate for the diagnosis of EGO. Also, the introduction of new disease-specific treatment with teprotumumab for TED has opened a new avenue in the management of this rare condition. It decreases reliance on steroids for treatment, essentially alleviating the side effects that come from the long-term use of steroids.
